# *In vitro* antibacterial effects of zinc oxide nanoparticles on multiple drug-resistant strains of *Staphylococcus aureus* and *Escherichia coli*: An alternative approach for antibacterial therapy of mastitis in sheep

**DOI:** 10.14202/vetworld.2018.1428-1432

**Published:** 2018-10-16

**Authors:** Myassar Alekish, Zuhair Bani Ismail, Borhan Albiss, Sara Nawasrah

**Affiliations:** 1 Department of Veterinary Clinical Sciences, Faculty of Veterinary Medicine, Jordan University of Science and Technology, Irbid 22110, Jordan; 2 Department of Applied Physical Sciences, Jordan University of Science and Technology, Irbid 22110, Jordan

**Keywords:** alternative therapy, antibiotics, mastitis, nanotechnology, sheep, zinc oxide

## Abstract

**Aim:**

The aim of the study was to evaluate the antibacterial effects of zinc oxide nanoparticles (ZnO-NPs) and its possible alternative use for the treatment for mastitis in sheep and to determine the minimum inhibitory concentration (MIC) and minimum bactericidal concentration (MBC) of ZnO-NPs against multidrug-resistant *Staphylococcus aureus* and *Escherichia coli* strains isolated from subclinical mastitis cases in sheep.

**Materials and Methods:**

A total of 50 pooled milk samples were collected from ewes with subclinical mastitis. Milk samples were cultured using standard laboratory techniques, and multidrug-resistant bacterial strains were determined using the Kirby–Bauer disk diffusion method. The MIC and MBC of ZnO-NPs were determined against isolated multidrug-resistant *S. aureus* and *E. coli* strains using microwell dilution method.

**Results:**

A total of 43 different bacterial isolates were recovered from milk samples of ewes affected with subclinical mastitis. Isolated strains of *S. aureus* and *E. coli* were found resistant to three or more common antibacterial agents and were used to determine the MIC and MBC of ZnO-NPs. The MIC and MBC values of ZnO-NPs were significantly lower for *S. aureus* than that for *E. coli*. The MIC and MBC of ZnO-NPs against *S. aureus* were 3.9 µg/ml and 7.81 µg/ml, respectively, while for *E. coli*, the MIC and MBC of ZnO-NPs were 31.25 µg/ml and 62.5 µg/ml, respectively.

**Conclusion:**

Results of this study indicate the potential antibacterial effects of ZnO-NPs against multidrug-resistant *S. aureus* and *E. coli* isolated from ovine subclinical mastitis at concentrations of 3.9 µg/ml and 31.25 µg/ml, respectively.

## Introduction

Conventional treatment of mastitis using antibiotics is costly and has led to the emergence of antimicrobial resistance (AMR) against most of the commonly used antibacterial agents [1-6]. Not only AMR is regarded as a serious threat to global public health and food security, but it also increases animal suffering and production losses [5,6].

Nanotechnology is the production of particles with dimensions of <100 nanometers [7-10]. Nanoparticles (NPs) have been shown to be a safe and effective alternative therapy against common infectious agents without driving antibiotic resistance in organisms [11-17]. The zinc oxide nanoparticles (ZnO-NPs) were found to improve the immune system in broilers and dairy cows when used as a feed supplement and to increase milk production in dairy cows [7-10]. Moreover, ZnO-NPs were found to possess antibacterial effects against a wide range of human and animal pathogenic bacteria with low toxicity to mammalian cells [12,13,16,17]. It was suggested that ZnO-NPs generate reactive oxygen species causing destructive oxidative stress to bacterial cells [18]. ZnO-NPs also were reported to induce an electrostatic disruption of cell membranes and leakage of intracellular material [18]. In addition, the soluble Zn2+ is believed to disrupt the metabolic activity of bacterial cells and therefore inhibit their growth [18].

In this study, the *in vitro* antibacterial effect of ZnO-NPs against multidrug-resistant *Staphylococcus aureus* and *Escherichia coli* isolates from subclinical mastitis in sheep was investigated by determining the minimum inhibitory concentration (MIC) and minimum bactericidal concentration (MBC) of ZnO-NPs.

## Materials and Methods

### Ethical approval

This study was performed in accordance with the International Animal Ethics Committee Guidelines and compliance with national laws and regulations. All experimental procedures were approved by the Jordan University of Science and Technology (JUST)-Animal Care and Use Committee.

### Animals

Lactating ewes were selected randomly from six different sheep flocks located in Northern Jordan. Ewes were 3-5 years of age and were in their 2^nd^ month of lactation. Before the inclusion in the study, all selected animals were subjected to a complete physical examination to determine their health status. The udder was further examined by palpation and milk was examined using the California mastitis test (CMT).

Ewes with clinical mastitis (hard and swollen udders and abnormal milk secretion such as watery, clotted, or bloody milk) were excluded from the study. Only apparently healthy ewes with positive CMT and normal looking udders were used in the study.

### Milk samples

Fifty pooled milk samples were collected from 50 lactating sheep affected with subclinical mastitis and were used in this study. Briefly, the teats were washed using warm water, and the teat ends wiped with 70% alcohol swabs before collecting the sample. The first 3-5 squirts of milk were discarded and approximately 10 ml of milk collected from both teats into sterile screw caps tubes. Samples were kept on ice during transportation to the laboratory. The bacteriological examination was performed within 2 h after sample collection.

### Bacterial isolation and identification

Approximately 0.01 ml of milk from each sample was cultured on 5% sheep Blood Agar Plates and MacConkey Agar Plates (Oxoid, UK) [19]. The agar plates were initially incubated at 37°C for 18-24 h. If no growth appeared after 24 h, incubation was extended for another 24 h before final judgment was taken. From growth-positive plates, isolates were sub-cultured on nutrient agar (HiMedia, India) plates at 37°C for 24 h to obtain pure cultures. Microorganisms were identified based on colony morphology, hemolysis characteristics on blood agar, and Gram-staining [19]. Gram-positive bacteria were then confirmed based on the results of the catalase test, oxidase test, DNase test, growth on mannitol salt agar, and Microbact 12S (Thermo Fisher Scientific, USA). Gram-negative bacteria were confirmed based on lactose fermentation on MacConkey Agar, Kligler Iron Agar, catalase test, oxidase test, and IMViC test (HiMedia Laboratories, India).

### Antibacterial sensitivity testing

All isolated bacteria were subjected to sensitivity testing against eight antibacterial agents using the Kirby–Bauer disk diffusion method on Mueller-Hinton (MH) agar plates [6]. Bacterial isolates were classified according to their sensitivity patterns against the major antibacterial groups. The antibacterial agents and their quantities in the disk were doxycycline 30 μg, enrofloxacin 5 μg, oxytetracycline 30 μg, penicillin G 10 μg, erythromycin 15 μg, streptomycin 10 μg, tetracycline 30 μg, and amoxicillin 10 μg. The inhibition zones around the disc were measured after 24 and 48 h of incubation at 37°C to classify the bacteria as susceptible, intermediate, or resistant using the breakpoints (diameters) previously published [6].

### Preparation of ZnO-NPs

ZnO-NPs were prepared in accordance to previously published methods [12]. Briefly, to a 500 ml of aqueous zinc sulfate heptahydrate, 1M sodium hydroxide solution was added at a rate of 0.2 ml/min with continuous stirring at room temperature until the pH level became 12. Then, ZnO-NPs were purified by washing with sterile distilled water and centrifugation at 2000× *g* for 15 min 3 times. The final product was then washed again with ethanol to remove any impurities. Finally, annealing of the solution containing ZnO-NPs was performed at 60°C using a hot plate to evaporate the liquid and separate the powdered ZnO-NPs.

**Figure-1:**
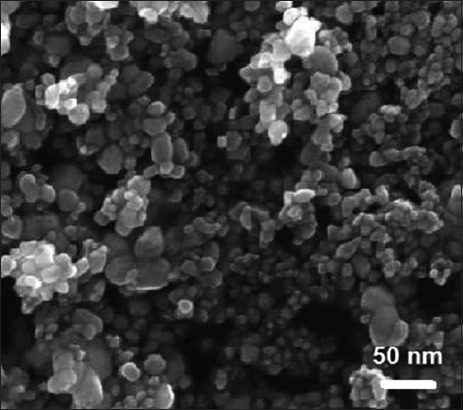
Scanning electron microscope images of zinc oxide nanoparticles (ZnO-NPs). ZnO-NPs were spherical with some aggregation.

**Figure-2:**
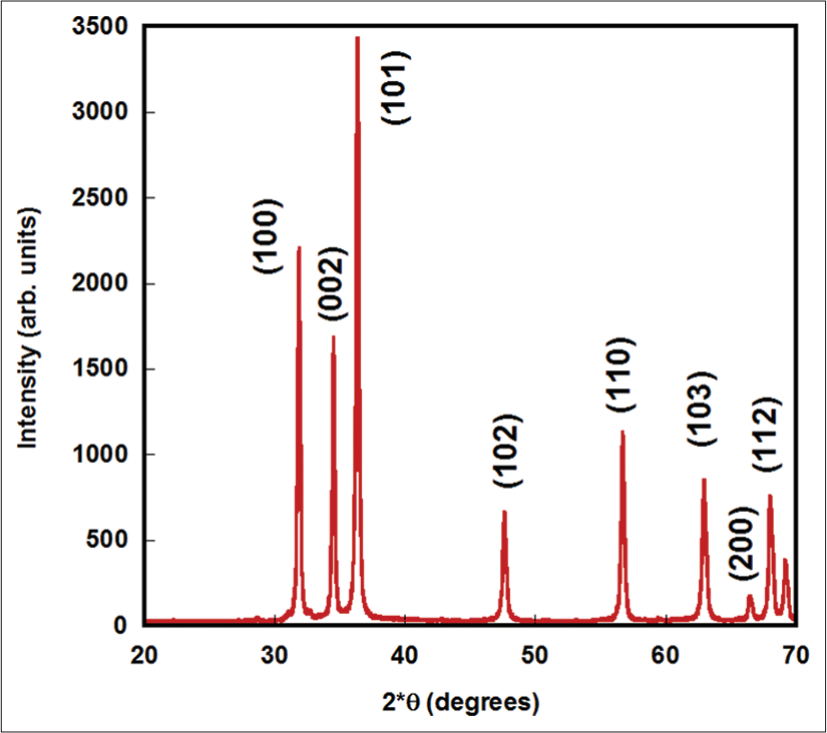
X-ray powder diffraction patterns of the zinc oxide (ZnO)-nanoparticles. All peaks indicate pure ZnO. No traces of other impurity phases were detected.

ZnO-NPs were obtained as a white powder with an average particle size of 30 nm and purity of above 99%. Field emission scanning electron microscope (JEOL, USA) [12] images of ZnO-NPs have shown that ZnO-NPs were almost spherical with some aggregation (Figure-1). Furthermore, the X-ray powder diffraction with CuKα (λ=1.5418 Å) radiation in the 2θ range of 20-70° and scan rate at 5°/min [12] (X-ray diffraction, Shimadzu 6000, Japan) patterns of the ZnO-NPs proved their crystalline nature and purity (Figure-2). No traces of other phase have been detected in these patterns. Miller indices were indexed for all major peaks in the pattern.

A stock solution of ZnO-NPs was prepared by adding distilled water to yield a final concentration of 1000 µg/ml. The stock solution was then stored at 4°C until used.

### Determination of MIC

MIC of ZnO-NPs of *S. aureus* and *E. coli* was performed using the microdilution method [20]. Briefly, overnight broth cultures of bacteria were suspended in MH broth with turbidity adjusted to 0.5 McFarland, resulting in a suspension containing approximately 108 CFU/ml [20]. To measure the MIC, 50 µl of MH Broth culture was poured into 12 wells of a 96-well microtiter plate. In the first well, 50 µl of the ZnO-NPs stock solution was added. A 2-fold dilution was then made to obtain different ZnO-NPs in each well (1000, 500, 250, 125, 62.5, 31.25, 15, 62, 7.81, 3.9, 1.95, 0.97, and 0.48 µg/ml). Then, 50 µl of the microbial suspension was added to each well. The microplate was then incubated at 37°C for 24 h, and the ZnO-NPs concentration in the well without visible growth of the bacterial cells was considered the MIC. A positive control contained MH Broth medium with tested bacterial concentrations and a negative control contained only inoculated broth were used in the study.

The MIC was defined as the least concentration of ZnO-NPs that visually inhibited the bacterial growth after 24 h of incubation [20]. The MIC was reported by observing the visual turbidity of the tubes before and after incubation. This was performed in five sets to confirm its value for the tested bacteria.

### Determination of MBC

To determine the MBC, 50 μl of broth was taken from all wells that showed no visible signs of growth/turbidity (MIC and higher dilutions) and streaked on MH agar plates [20]. The plates were then incubated at 37°C for 24 h. The MBC was defined as the least concentration of ZnO-NPs that prevented the growth of the bacteria on antibiotic-free culture media [20].

### Statistical analysis

To apply statistical analysis, both the MIC and MBC experiments were performed in five sets. In the MIC experiment, a numerical value of 0 was assigned to wells with no inhibition of bacterial growth and a numerical value of 1 was assigned to wells with inhibition of bacterial growth. In the MBC experiment, the numerical value 0 was assigned, if bacterial growth was observed, and 1 if no bacterial growth was observed on the agar plate. The data were then analyzed using descriptive statistics including mean and standard deviation. One-way ANOVA and non-paired t-test were used to analyze MIC and MBC of ZnO-NPs against *S. aureus* and *E. coli*. Values were considered significant at p<0.01. Statistical analysis was performed using IBM SPSS Statistics for Windows, Version 23.0 (IBM Corp., NY USA).

## Results

A total of 43 different bacterial isolates were recovered. The majority of isolates (93%) were classified as coagulase-negative staphylococci. Only two isolates were identified as *S. aureus* and one isolate was identified as *E. coli*. Both strains of *S. aureus* and *E. coli* that were isolated in this study were found resistant to five and six different antibacterial agents, respectively.

The MIC and MBC values of ZnO-NPs were significantly lower for *S. aureus* than that for *E. coli* (Table-1). When the growth of inoculated bacteria was assessed at different concentrations of ZnO-NPs after 24 h, the MIC and MBC of ZnO-NPs against isolated *S. aureus* were 3.9 µg/ml and 7.81 µg/ml, respectively. For *E. coli*, the MIC and MBC of ZnO-NPs were 31.25 µg/ml and 62.5 µg/ml, respectively.

**Table-1:** The MIC and MBC values (µg/ml) of ZnO-NPs against *S. aureus* and *E. coli* strains isolated from ewes affected with subclinical mastitis using microdilution method.

Bacterial isolate	MIC	MBC
*S. aureus*	3.9	7.81
*E. coli*	31.25	62.5

MIC=Minimum inhibitory concentration, MBC=Minimum bactericidal concentration, ZnO-NPs=Zinc oxide nanoparticles, *S. aureus=Staphylococcus aureus, E. coli=Escherichia coli*

Statistical analysis of MBC against *S. aureus* for different concentrations showed that the optimum MIC was obtained at 7.81 µg/ml which confirms that the MIC and MBC of ZnO-NPs against *S. aureus* were effective at 3.9 µg/ml and 7.81 µg/ml, respectively. For *E. coli*, statistical analysis of MBC for different concentrations showed that the optimum MIC was obtained at 31.25 µg/ml which confirms that the MIC and MBC of ZnO-NPs against *E. coli* were effective at 31.25 µg/ml and 62.5 µg/ml, respectively.

## Discussion

The antibacterial sensitivity test results in this study indicate the widespread resistance among *S. aureus* and *E. coli* isolates against most commonly used antibacterial agents in Jordan. All isolated strains of *S. aureus* and *E. coli* were found resistant to five and six different antibacterial agents, respectively. Recent studies involving dairy cows and sheep in Jordan indicated an alarmingly increasing incidence of AMR [6,21,22]. Multidrug resistance patterns of *S. aureus* and *E. coli* similar to the one reported here have been reported previously in many parts of the world [23,24].

Although it has been used in biomedical and therapeutic research in human medicine, the antibacterial activities of ZnO-NPs have not been studied against common mastitis pathogens in animals. Therefore, this is the first study to investigate the antibacterial effects of ZnO-NPs against common and multidrug-resistant mastitis pathogens in sheep. The antibacterial effects of ZnO-NPs were determined by measuring the MIC and MBC against multidrug-resistant *S. aureus* and *E. coli* strains isolated from subclinical mastitis in sheep.

In this study, the MIC and MBC of ZnO-NPs against *S. aureus* and *E. coli* were 3.9 µg/ml and 7.81 µg/ml, respectively. For *E. coli*, the MIC and MBC of ZnO-NPs were 31.25 µg/ml and 62.5 µg/ml, respectively. These results were similar to previously reported findings [14,17]. Moreover, our results are in agreement with previous results that indicated that Gram-positive bacteria were more sensitive than Gram-negative bacteria to ZnO-NPs [14,17]. In general, it was established that the inhibitory efficacy of ZnO-NPs is highly dependent on its concentration and size [25].

## Conclusion

In this study, the antibacterial effects of ZnO-NPs against multidrug-resistant *S. aureus* and *E. coli* were investigated for the 1^st^ time as an approach for antibacterial therapy of mastitis in sheep. Both *S. aureus* and *E. coli* strains were susceptible to ZnO-NPs at concentrations of 3.9 µg/ml and 31.25 µg/ml, respectively. ZnO-NPs could be formulated in a suitable intramammary applicator for use in the treatment of clinical and subclinical mastitis caused by *S. aureus* and *E. coli* in sheep. However, case-controlled and field efficacy studies are still warranted at this stage to evaluate its efficacy and safety.

## Authors’ Contributions

MA designed the study and supervised fieldwork. ZBI performed manuscript writing and data interpretation. BA prepared ZnO-NPs. SN performed field and laboratory work. All authors read and approved the final manuscript.
